# Amoxicillin‐Induced Drug‐Induced Liver Injury Superimposed on Acute Hepatitis C Infection in a Patient With Hurler Syndrome: A Diagnostic Challenge Assessed by the Updated RUCAM

**DOI:** 10.1155/crhe/5815563

**Published:** 2026-06-15

**Authors:** Archit Garg, Prosper Seshie, Abhishek Chouthai, Sahil Raval, Aadhithyaraman Santharaman, Adil Manzoor, Arkady Broder

**Affiliations:** ^1^ Internal Medicine Department, Saint Peter’s University Hospital, New Brunswick, New Jersey, USA, saintpetershcs.com; ^2^ Gastroenterology Department, Saint Peter’s University Hospital, New Brunswick, New Jersey, USA, saintpetershcs.com

**Keywords:** DILI, drug-induced liver injury, hepatitis C, Hurler syndrome, Roussel Uclaf Causality Assessment Method, RUCAM, updated RUCAM 2016

## Abstract

**Background:**

Drug‐induced liver injury (DILI) refers to hepatotoxicity caused by conventional chemical drugs or xenobiotics, whereas herb‐induced liver injury (HILI) is attributed to herbal and dietary supplements. Both these conditions pose diagnostic challenges, particularly when concurrent etiologies such as acute viral hepatitis are present. Hurler syndrome (mucopolysaccharidosis Type I) causes hepatocyte and Kupffer cell vacuolization and can predispose to DILI. Early diagnosis is critical given the high fatality rates associated with DILI.

**Case Report:**

We present a case of a 28‐year‐old male with Hurler syndrome who presented with acute onset of nausea, vomiting, and jaundice. Liver function tests (LFTs) revealed markedly elevated liver enzymes. Serological workup identified newly acquired acute hepatitis C virus (HCV) infection. The patient had recent amoxicillin use and was taking hibiscus tea daily. Causality assessment using the updated Roussel Uclaf Causality Assessment Method (RUCAM) of 2016 yielded a score of 6 (probable DILI). Liver biopsy confirmed DILI. The patient showed clinical improvement with N‐acetylcysteine and corticosteroids, with progressive normalization of liver enzymes.

**Conclusions:**

This case highlights the importance of differentiating DILI from acute viral hepatitis: strong clinical suspicion, temporal relation with offending drug, liver biopsy, and treatment response assessment. Clinicians should have a high index of suspicion for DILI even in the presence of concurrent acute HCV infection, especially in patients with underlying hepatic dysfunction such as Hurler syndrome in our case.

## 1. Introduction

Hurler syndrome or mucopolysaccharidosis type I (MPS I) or gargoylism is one of the eleven mucopolysaccharidoses disorders [1]. It is characterized by the deficiency of alpha‐L‐iduronidase enzyme which results in accumulation of glycosaminoglycans/GAG (heparin sulfate and dermatan sulfate) in the cells eventually leading to cell dysfunction and apoptosis. Hurler syndrome, the most severe phenotypic form of MPS I, encompasses early onset of clinical manifestations which if not identified and diagnosed leads to high incidence of first decade mortality without treatment [2]. Coarsening of the facial features (including frontal bossing, flat nasal bridge, enlarged lip, and widely spaced eyes), recurrent respiratory tract infections, otitis media, hearing disturbances, corneal clouding, kyphosis, and neurocognitive developmental delay are some of the early disease manifestations of Hurler syndrome [3]. Cardiomyopathies with early onset heart failure, obstructive airway disease, and recurrent respiratory tract infections contribute toward early mortality [4]. Symptomatic treatment, enzyme replacement therapy, and early bone marrow transplant increase life expectancy and contribute toward somewhat increased quality of life [5].

The GAG deposition in Hurler Syndrome causes vacuolization of hepatocytes and Kupffer cells, eventually leading to hepatomegaly, hepatic dysfunction, and could even progress to hepatic fibrosis [6, 7]. The progressive nature of Hurler Syndrome means that liver function can progressively deteriorate. As the liver plays a significant role in the body’s metabolic processes, especially metabolizing drugs and toxins, hepatic dysfunction can contribute toward altered drug metabolism, transport, and clearance of foreign substances, and hence organ failure through drug toxicity [8, 9]. Drugs, herbal supplements, or xenobiotics causing liver injury manifested by abnormal liver function tests (LFTs) or liver dysfunction (imaging or biopsy proven) after excluding other etiologies is known as drug‐induced liver injury (DILI) [10].

DILI accounts for around 14–19 cases per 100,000 population [11]. It is the most common cause of acute liver failure (ALF) with a case fatality rate of 10%–50% in the western world [12]. DILI can be intrinsic/nonidiosyncratic (predictable and dose‐dependent, e.g., acetaminophen) or idiosyncratic (unpredictable, more common, e.g., antibiotics, analgesics, and chemotherapy drugs) [13]. Genetic, epigenetic and environmental factors play an important predisposing role in DILI. Hepatic dysfunction, chronic liver disease, HIV, and obesity can predispose individuals to DILI [14]. Here, we present a unique case of DILI in a patient with Hurler Syndrome and the diagnostic challenges in coming to this diagnosis. With newly diagnosed hepatitis C virus (HCV) infection, elevated LFTs can be misleading and the diagnosis of DILI can be challenging and often overlooked.

## 2. Case Description

A 28‐year‐old male presented to the emergency department with a chief complaint of nausea and vomiting for 1 week. Symptoms started suddenly, were worse with food intake, and vomitus was nonbloody and nonbilious. Over the week, symptoms worsened, and he noticed yellowish discoloration of the skin and eyes, dark‐colored urine, and dull abdominal discomfort (more in the right upper quadrant), prompting him to seek hospital evaluation. He denied fever, chills, outside food consumption, recent travel, illicit drug use, sick contacts, or similar symptoms in the past. His past medical history was significant for Hurler Syndrome confirmed by genetic testing in 2009; since then, he had been on weekly enzyme supplementation with laronidase infusions, with consistently normal LFTs documented on all prior visits up to three months before this presentation. His social history was positive for occasional alcohol consumption (2 cans of beer twice a week for 8 years, with the last drink 4 days prior to admission).

Vital signs on admission were within normal limits, and the patient was not in any apparent distress. General appearance was remarkable for short stature, flat face, depressed nasal septum, prominent broad budging forehead (frontal bossing), wide‐spaced eyes, corneal clouding, generalized icterus (sclera, oral mucosa and skin) and clubbing of nails. Cardiac examination showed a systolic murmur in the aortic and mitral region. Abdominal examination revealed diffuse tenderness in all quadrants. Laboratory testing showed a normal complete blood count. However, comprehensive metabolic panel demonstrated markedly abnormal LFTs (ALP [Alkaline phosphatase] 203 U/L, aspartate aminotransferase }AS]) 1609 U/L, alanine aminotransferase [ALT) 2019 U/L, total bilirubin 9.3 mg/dL, and direct bilirubin 4.4 mg/dL]. For reference, his most recent LFTs 3 months prior were entirely within the normal range (ALP 93 U/L, AST 25 U/L, ALT 23 U/L, and total bilirubin 0.8 mg/dL), confirming the acute nature of this hepatic event. INR was 1.16, and since the patient was not encephalopathic, ALF was excluded. With such markedly elevated LFTs, possible differentials included infectious or alcoholic hepatitis, obstructive pathology (including cholelithiasis/choledocholithiasis), or autoimmune hepatitis. Right upper quadrant ultrasound and CT abdomen were unremarkable, ruling out obstructive pathology. Serum alcohol levels were undetectable, and the absence of alcohol binges with AST/ALT < 1 made alcoholic hepatitis unlikely.

On reviewing the results with the patient and probing further for medication history, the patient disclosed that he had taken amoxicillin for approximately 5–7 days for an upper respiratory tract infection 3 weeks prior and had taken 8 tablets of acetaminophen (500 mg each) over 2 days (total 4 g), 2 weeks prior, for headache. He denied consuming herbal supplements but reported occasional hibiscus tea consumption. He also reported being sexually active with males, using barrier contraception infrequently. Based on this information, further serologic and biochemical workup was obtained. Serum salicylate and acetaminophen levels, antismooth muscle antibody, and antinuclear antibody were all negative. Epstein–Barr Virus (EBV), Cytomegalovirus (CMV), Herpes Simplex Virus (HSV) IgM, and Human Immunodeficiency Virus (HIV) were negative. Viral hepatitis panel demonstrated hepatitis A immunity and positive hepatitis C antibodies. Despite normal hemoglobin (13.8 g/dL), the patient had elevated serum iron (248 mcg/dL) and ferritin (1090 ng/mL). The elevated ferritin and serum iron in this context were interpreted as acute‐phase reactants associated with hepatocellular necrosis (as seen in acute hepatitis), rather than indicative of primary hemochromatosis.

At this point, differentials narrowed to acute viral hepatitis C, DILI, or hemochromatosis. The constituents of the laronidase bag (laronidase, sodium chloride, monobasic sodium phosphate monohydrate, dibasic sodium phosphate heptahydrate, and Polysorbate 80) were reviewed; none have been implicated in DILI, and the patient had been receiving this infusion for years without prior LFT abnormalities, making it an unlikely contributor. A positive HCV RNA viral load confirmed acute HCV infection. However, given the temporal relationship between amoxicillin and acetaminophen use and the onset of elevated LFTs, and given the high fatality rates associated with DILI, causality assessment using the updated Roussel Uclaf Causality Assessment Method (RUCAM) of 2016 was performed. The R ratio ([ALT/ULN]/[ALP/ULN]) was calculated as (2019/55)/(203/130) = 36.7/1.56 = 23.5, consistent with a hepatocellular pattern. The updated RUCAM score was calculated as 6 (probable DILI) based on the time to onset, course of LFTs after drug withdrawal, known hepatotoxic risk of amoxicillin, and exclusion of competing etiologies. Given these findings, the patient was started on N‐acetylcysteine (NAC) (150 mg/kg over 1 h, followed by 50 mg/kg over 4 h, then 100 mg/kg over 16 h intravenously) as a precautionary measure.

**FIGURE 1:**
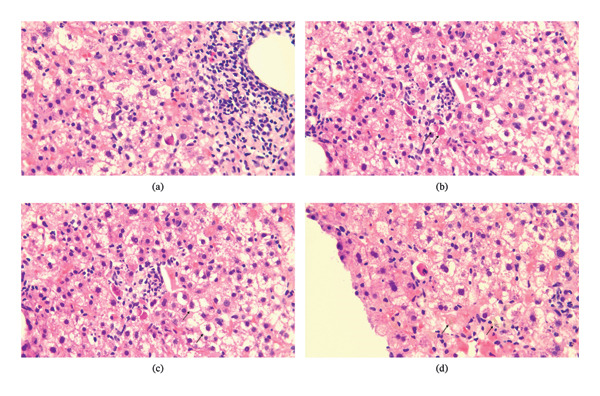
Liver biopsy results suggestive of DILI. (a) Periportal infiltrate, predominantly lymphocytes and eosinophils. (b) Apoptotic bodies (arrows). (c) Ceroid laden macrophages (arrows). (d) Feathery degeneration of hepatocytes (arrows).

Liver enzymes trended downward, and the patient began to improve clinically. A liver biopsy was performed for histopathological characterization. After 3 days, due to symptomatic improvement and declining LFTs, methylprednisolone 40 mg intravenously daily was initiated for suspected DILI. Liver biopsy results (Figure 1a, b, c, d) showed acute cholestatic hepatitis with eosinophilic and lymphocytic periportal infiltrates, apoptotic bodies, ceroid‐laden macrophages, and feathery degeneration of hepatocytes—findings most consistent with DILI. Notably, the histological pattern particularly the prominent eosinophilic infiltration and cholestatic features was suggestive with DILI than acute HCV hepatitis, which more commonly manifests with a lymphocytic portal infiltrate without significant eosinophilia or cholestasis. After 3 days of intravenous steroids with continuous improvement in LFTs, the patient was transitioned to oral prednisone (40 mg daily, tapered by 10 mg per week) and discharged with advice to avoid hibiscus tea, acetaminophen, and antibiotics, and to consult gastroenterology before initiating any new medications.

At follow‐up with gastroenterology, the patient reported symptomatic improvement and complete normalization of LFTs at the 45‐day follow‐up visit. His HCV viral load at the time of discharge was 5,820,000 IU/mL. He was initiated on direct‐acting antiviral (DAA) therapy with glecaprevir/pibrentasvir for 8 weeks with subsequent HCV viral load being undetectable after 4 months.

**FIGURE 2:**
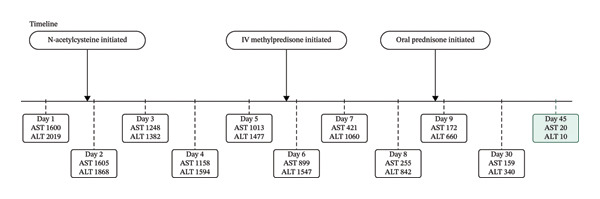
Trend line of liver enzymes.

Trend line of liver enzymes is shown in Figure 2.

## 3. Discussion

DILI constitutes any liver injury that occurs due to a prescribed or over‐the‐counter medication, or herbal and dietary supplements, with clinical manifestations ranging from asymptomatic elevation of liver enzymes to full‐blown ALF [14]. It is essential to distinguish DILI, caused by conventional chemical drugs from HILI, which results from herbal medicines and dietary supplements. DILI can be intrinsic/nonidiosyncratic (dose‐dependent, predictable, e.g., acetaminophen) or idiosyncratic (variable latency, unpredictable, e.g., amoxicillin–clavulanate, NSAIDs, ketoconazole, nitrofurantoin, sulfonamides, and isoniazid) [15, 16]. A prospective study in the United States of America showed that idiosyncratic DILI and nonidiosyncratic (acetaminophen‐induced) DILI contributed to 39% and 13% of ALF cases, respectively [15]. Early identification and management are therefore of paramount importance.

A variety of genetic, epigenetic, and environmental factors play a role in the increased risk of DILI. Female sex, advanced age, HIV infection, obesity, and chronic liver disease all predispose to DILI [14]. Certain HLA genotypes and polymorphisms in cytochrome P450 enzymes have been strongly associated with DILI; for example, HLA Class II allele DRB1∗15:01 is associated with amoxicillin–clavulanate DILI, and HLA B∗57:01 and B∗57:03 with flucloxacillin DILI [17]. Pre‐existing liver pathologies including hepatitis B or C and metabolic dysfunction–associated steatotic liver disease (MASLD) can augment inflammatory responses to drug exposure and predispose to DILI, although the evidence remains contentious [18]. There is no direct evidence that Hurler syndrome predisposes to DILI. However, hepatic dysfunction with vacuolization of hepatocytes and Kupffer cells causing hepatomegaly has been documented in Hurler Syndrome [6, 7]. Kupffer cells have a dual, controversial role in DILI: Some studies report that Kupffer cells secrete proinflammatory cytokines (IL‐6 and TNF‐alpha) and reactive oxygen species that exacerbate early hepatic inflammation [19], while others demonstrate a protective Kupffer cell role, with animal models showing that reduced IL‐10 secretion increases susceptibility to DILI [19]. In the present case, obesity, concurrent HCV infection, and baseline hepatic structural vulnerability in the setting of Hurler Syndrome may have collectively predisposed the patient to DILI.

Among medications implicated in DILI, amoxicillin–clavulanate is more commonly cited than amoxicillin alone; the incidence of DILI with amoxicillin alone is approximately 0.3 per 10,000 prescriptions, compared to 1.7 per 10,000 for amoxicillin–clavulanate [20–22]. The hepatoprotective versus hepatotoxic role of hibiscus (*Hibiscus sabdariffa*) is debated: Some studies demonstrate amelioration of acetaminophen‐induced hepatotoxicity [23], while others document inhibition of CYP3A4 and CYP1A2 enzymes, potentially altering amoxicillin metabolism [24]. In the present case, amoxicillin alone, amoxicillin in combination with hibiscus tea, or the triad of amoxicillin, hibiscus tea, and acetaminophen (4 g over 48 h) may all have contributed to DILI.

The diagnosis of DILI remains challenging and requires a high degree of clinical suspicion. The updated RUCAM of 2016 represents the most widely validated tool for systematic causality assessment of DILI [25]. This assessment incorporates time to onset, course after drug withdrawal, risk factors, comedication, nondrug causes, prior hepatotoxicity data, and response to readministration [25]. In our case, the updated RUCAM 2016 score was 6, classifying the diagnosis as “probable DILI.” This structured assessment is critical and currently represents the gold standard for DILI causality evaluation. To date, over 81,856 DILI cases worldwide have been assessed using RUCAM through mid‐2020, underscoring its global clinical utility. The R ratio confirmed a hepatocellular pattern of injury (*R* = 23.5), consistent with amoxicillin‐induced hepatocellular DILI. The hepatocellular pattern of DILI is associated with the worse prognosis compared to cholestatic or mixed patterns [14]. Excluding alternative causes of liver injury including viral hepatitis (HAV, HBV, HCV, HEV, EBV, and CMV), MASLD, alcoholic hepatitis, autoimmune hepatitis, Wilson’s disease, hemochromatosis, Alpha‐1 antitrypsin deficiency, and celiac disease remains essential [14]. Notably, elevated serum ferritin in this case likely represents an acute‐phase response to hepatocellular necrosis in acute hepatitis rather than a primary iron overload state. Liver biopsy showing cholestatic hepatitis with prominent eosinophilic infiltration, the most common histological finding in DILI, as identified by meta‐analysis [26], further supports the DILI diagnosis.

Treatment of DILI centers on prompt withdrawal of the offending agent. NAC has demonstrated benefit in idiosyncratic nonacetaminophen DILI, particularly in cases progressing toward ALF [14, 27]. Corticosteroids have been shown to lower liver enzyme levels, improve symptoms, and enhance patient survival in selected DILI cases [28, 29]. In this patient, early NAC administration followed by methylprednisolone resulted in rapid clinical improvement and progressive normalization of LFTs.

Drug‐induced autoimmune hepatitis (DIAIH) is a distinct DILI variant which is characterized by features overlapping with autoimmune hepatitis [30]. However, the absence of positive autoimmune serologies (antinuclear antibody or antismooth muscle antibody), the temporal relationship with drug exposure, and the lack of relapse after corticosteroid discontinuation argue against DIAIH and support a diagnosis of classic DILI. The concurrent acute HCV infection in this case created significant diagnostic complexity. A retrospective study found that 1.5% of suspected DILI cases were attributable to acute HCV infection [31], highlighting the importance of comprehensive viral hepatitis workup in all patients with suspected DILI. In the present case, the histological features of the liver biopsy and the clinical response to corticosteroids supported DILI as the primary diagnosis.

## 4. Conclusions

This case report presents a diagnostically challenging scenario involving amoxicillin‐induced (in combination with hibiscus tea) DILI superimposed on acute hepatitis C infection in a patient with Hurler Syndrome. Causality assessment using the updated RUCAM of 2016 yielded a score of 6 (probable DILI), providing structured, evidence‐based support for the diagnosis. Histopathological findings (acute cholestatic hepatitis with prominent eosinophilic infiltration) were more consistent with DILI than acute viral hepatitis C, and clinical response to NAC and corticosteroids further supported this diagnosis.

Clinicians must maintain a heightened index of suspicion for DILI even when concurrent viral hepatitis is identified. Systematic application of the updated RUCAM 2016 is essential to avoid diagnostic misclassification. In patients with Hurler Syndrome, baseline hepatic structural vulnerability may amplify susceptibility to drug‐induced hepatotoxicity. Furthermore, comprehensive longitudinal follow‐up is warranted to monitor HCV clearance or progression and to guide antiviral therapy decisions. This case adds to the growing literature underscoring that DILI and acute HCV infection can coexist and that multidisciplinary evaluation with integrating clinical data, causality scoring, histopathology, and treatment response is indispensable for accurate diagnosis and management.

**Nomenclature**
ALP: Alkaline phosphatase
ALT: Alanine aminotransferase
AST: Aspartate aminotransferase
CMV: Cytomegalovirus
DILI: Drug‐induced liver injury
EBV: Epstein–Barr Virus
HCV: Hepatitis C virus
HIV: Human Immunodeficiency Virus
HSV: Herpes Simplex Virus
INR: International normalized ratio
LFTs: Liver function tests
MASLD: Metabolic dysfunction–associated steatotic liver disease
MPS I: Mucopolysaccharidosis Type I
NAC: N‐acetylcysteine
RUCAM: Roussel Uclaf Causality Assessment Method
ULN: Upper limit of normal

## Author Contributions

Archit Garg, Prosper Seshie, and Adil Manzoor: conception of the study idea. Archit Garg, Prosper Seshie, Abhishek Chouthai, Aadhithyaraman Santharaman, and Sahil Raval: drafting of the initial manuscript. Arkady Broder and Adil Manzoor: reviewing, editing, and final approval of the manuscript.

## Funding

The authors have nothing to report.

## Consent

Patient consent was obtained for publication of the case report.

## Conflicts of Interest

The authors declare no conflicts of interest.

## Data Availability

Data sharing is not applicable to this article as no datasets were generated or analyzed during the current study.
